# HIV-1 patients may harbor viruses of different phylogenetic subtypes: implications for the evolution of the HIV/AIDS pandemic.

**DOI:** 10.3201/eid0103.950303

**Published:** 1995

**Authors:** D Pieniazek, L M Janini, A Ramos, A Tanuri, M Schechter, J M Peralta, A C Vicente, N K Pieniazek, G Schochetman, M A Rayfield

**Affiliations:** National Center for Infectious Diseases, Centers for Disease Control and Prevention, Atlanta, Georgia, USA.


### HIV-1 Patients May Harbor Viruses of Different Phylogenetic Subtypes: Implications for the Evolution of the HIV/AIDS Pandemic

The virus variants isolated from HIV-infected persons worldwide share remarkable diversity, especially in the envelope glycoprotein, gp120. Phylogenetic studies have clustered HIV-1 isolates into eight subtypes (A-H). Nevertheless, even within a single infected person, HIV is present as a "quasispecies," or a swarm of closely related variants. This genetic diversity, which in the case of HIV-1 accumulates at a rate of approximately one nucleotide substitution per genome per replication cycle, gives the virus an enormous flexibility to respond to a wide array of in vivo selection pressures. As a consequence, drug-resistant and immunologic escape mutants are rapidly generated in infected persons through all stages of infection. On a global scale, the HIV pandemic is recognized as consisting of many separate epidemics, each with characteristic geography, affected populations, and predominant viral strain type. With an estimated 15 million infected persons, the geographic distribution of viral subtypes is becoming more dispersed, and these demarcations are further confounded by growing evidence of mixed infections.

The epidemic emergence of mixed heterotypic infections with HIV-1 and HIV-2 variants has been recognized for some time in the geographic areas where both types of viruses are present. We reported these infections in Côte d'Ivoire and Brazil (1, 2); they have also been reported from India (3). In contrast, homotypic mixed infections of distinct HIV-1 variants have only recently been suggested by the presence of broadly reactive sera and evidence of HIV recombinants from geographic regions in which multiple HIV-1 subtypes are circulating. Dual HIV-1 infection in two patients from Thailand has been demonstrated by viral DNA sequence analysis (4).

As the HIV-1 pandemic has grown, the simultaneous presence of multiple subtypes in a region has become common. As a consequence, an increased frequency of HIV-1 mixed infections could be expected. Thus, there is a need to estimate the prevalence and geographic distribution of this type of infection. Sequence analysis of HIV proviral DNA has been the method of choice to characterize HIV genetic diversity. However, because even relatively limited sequence determinations of small polymerase chain reaction (PCR) fragments are time consuming and very labor-intensive, this method is not particularly practical for large-scale molecular epidemiologic studies. To address this problem, we have developed a genetic method based on restriction site polymorphism to screen for homotypic HIV-1 mixed infections within infected populations. The concept of this assay is based on the observed correlation between the restriction maps of HIV-1 isolates with their phylogenetic classification, which is based on the sequence data. Thus, certain restriction enzymes may be used to predict the phylogroup of HIV-1 infected samples. The differences in electrophoretic mobility of endonuclease digestion products result from restriction site polymorphisms in the selected region of the HIV-1 genome and allow for quick recognition of the distinct phylogenetic subtypes. A 297 bp pol fragment spanning the entire viral protease gene is used for our analysis. The viral gene is amplified by nested PCR using DNA templates from uncultured peripheral blood mononuclear cells (PBMC) or virus culture. Preliminary classification of HIV-1 strains to well defined subtypes A, B, C, D, and F is done by sequential endonuclease restriction analysis. AluI restriction polymorphism in a PCR-amplified protease gene segregates viral strains into two groups: subtypes B and D belong to one group, and subtypes A, C, and F to another (Figure 1A). Further differentiation of HIV-1 subtypes within those two groups is accomplished by analysis of HinfI, BclI, MaeI, SpeI, and ScaI restriction enzyme digestion patterns of the protease gene (Pieniazek et al., manuscript in preparation). The electrophoretic migration patterns visualized by ethidium bromide staining or by radiolabeled probes are then determined on a 10% acrylamide gel. In single infections, a single restriction pattern is detected, whereas in multiple infections involving HIV-1 strains of distinct subtypes, complex digestion patterns are observed in infected persons. As an example, in Figure 1A, we present three distinct AluI restriction patterns of the protease gene that are characteristic for single infections by viruses of subtypes A, C, and F (pattern #1) and by subtypes B and D (patterns #2 and #3). In Figure 1B, we show a typical combination of two distinct AluI restriction patterns (#1 and #2) found in a patient infected with two viral strains of subtypes F and B. Basing our analysis on the conserved protease gene region, we should detect most HIV-1 strains; however, some highly divergent isolates could escape PCR amplification as a result of primer mismatches. Moreover, since a single nucleotide substitution could either generate or destroy a restriction site, sequence analysis remains the ultimate tool in identifying variants of multiple infections. Nevertheless, this assay can be conveniently applied to screen a large number of samples.

By using this method, we have screened HIV-1 proviral DNA from 208 specimens collected from countries in South America, Africa, and Asia where HIV-1 strains of distinct subtypes are found. We observed the simultaneous presence of two distinct digestion patterns in PCR amplified protease gene (Figure 1B) in 31 samples; our observation suggests superinfection with HIV-1 strains of distinct origin. To eliminate the potential for laboratory cross-contamination, we analyzed the restriction patterns of the protease gene from multiple aliquots of the patient's PBMC. In addition, the analysis was repeated on DNA from a second collected blood sample from each of the patients. The analyses for the first five of 31 patients were completed, and data are summarized here (details are in Janini et al., manuscript in preparation). Sequence and phylogenetic analysis of the viral protease gene (Figure 2) in PBMC from those five patients confirmed dual infections caused by HIV-1 strains of subtypes B and F in one person (Br5), subtypes F and D in another patient (Br22), and subtypes C and D in a married couple (Br19 and 20). Moreover, in the child (Br30) of this couple, two distinct AluI digestion patterns were also found; the major HIV-1 strain clustered among subtype C viruses of the parents. The minor strain of this child is likely to represent subtype D, but there was not sufficient material for cloning and further sequencing of this strain.

Detection of naturally occurring heterotypic and homotypic multiple infections may have important implications for immunotherapies because infection with one HIV subtype may not fully protect against subsequent superinfections with distinct HIV strains. However, we do not know if the acquisition of viruses in the dually infected adult patients was sequential or simultaneous. Nevertheless, the consequences of mixed infections may profoundly affect the ability of the virus to change and may modify the direction of the pandemic through altered patterns of viral pathogenesis, increased genetic variation through recombination, and the generation of

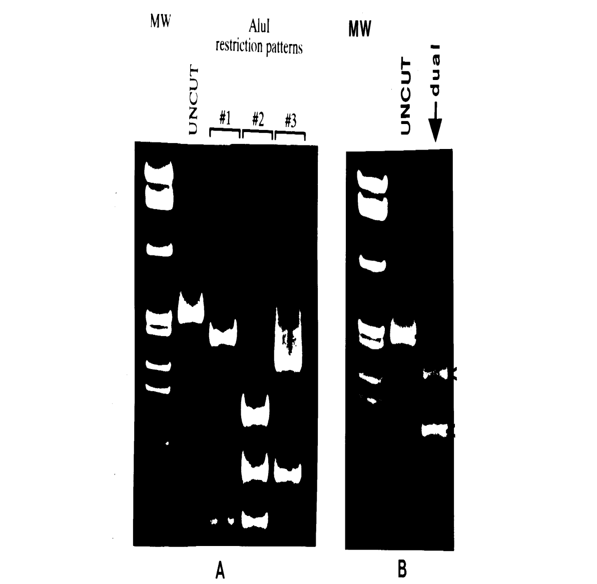

**Figure 1.**
A. Three distinct AluI digestion patterns of PCR amplified protease gene representing single HIV-1 infections by viral strains of subtypes A, C, and F (pattern #1), and subtypes B and D (patterns #2 and #3).
B. The presence of two distinct AluI digestion patterns (#1 and #2) of the protease gene in PBMC of the patient dually infected by viral strains of subtypes F and B (lane 3). Arrows indicate diagnostic fragments detected by hybridization with the radioactive probe (2). MW represents molecular weight markers—ΦX174 RF DNA, HaeIII digest.

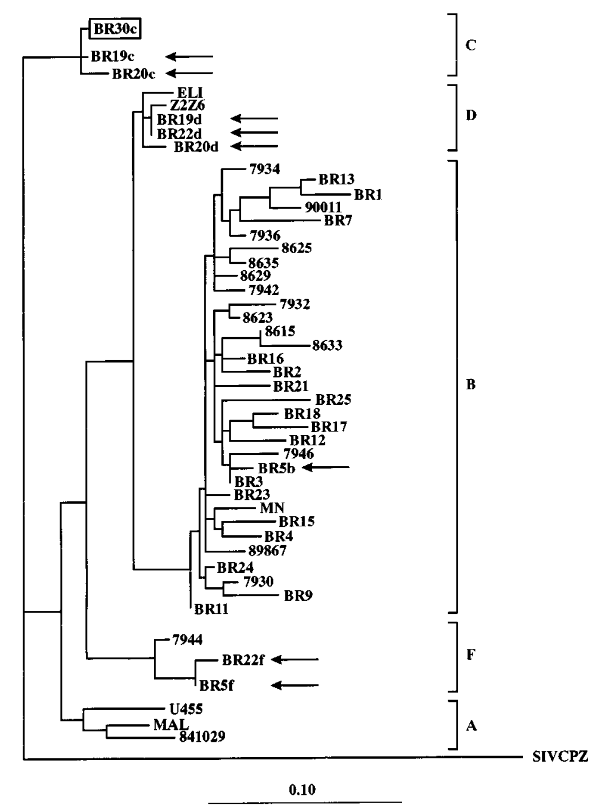

**Figure 2.**
Phylogenetic classification of HIV-1 strains in dually infected patients. HIV-1 sequences from dual infections (Br5, 19, 20 and 22 ) are indicated by arrows, and the major strain in the infected child (Br30) is boxed. The tree was constructed on the basis of the DNA sequences of the protease gene by using the maximum likelihood method with the fastDNAml program (6). SIV-cpz protease sequence was used as an outgroup. The distinct HIV-1 subtypes are delineated. The scale bar shows the ratio of nucleotide substitutions for given horizontal branch length. Vertical distances are for clarity only.

pseudotype virions, including phenotypically mixed virus particles. It is to be anticipated that such events would ultimately broaden the cellular tropism for HIV and mandate the designed polyvalent immunotherapies. Finally, our data together with recently published genetic analysis for HIV-1 and HIV-2 (5) suggest that multiple homotypic infections with divergent HIV strains may be more common than previously thought. The screening assay described here will be useful in estimating incidences of such HIV-1 infections. We believe that this information is crucial for both evaluating the pandemic and developing intervention strategies.

**Danuta Pieniazek,* Luiz M. Janini,*† Artur Ramos,*† Amilcar Tanuri,† Mauro Schechter,‡ Jose M. Peralta,† Anna C.P. Vicente,§ Norman J. Pieniazek,* Gerald Schochetman,* and Mark A. Rayfield***

*National Center for Infectious Diseases, Centers for Disease Control and Prevention, Atlanta, Georgia, USA; †Universidade Federal do Rio de Janeiro, and ‡Hospital Clementino Fraga Filho, and §Instituto Oswaldo Cruz, Rio de Janeiro, Brazil

### References

1. Rayfield M, De Cock K, Heyward W, et al. Mixed human immunodeficiency virus (HIV) infection in an individual: demonstration of both type 1 and type 2 proviral sequences by using polymerase chain reaction. J Infect Dis 1988:158:1170-6.
2. Pieniazek D, Peralta JM, Ferreira JA, et al. Identification of mixed HIV-1/HIV-2 infections in Brazil by polymerase chain reaction. AIDS 1991;5:1293-9.
3. Grez M, Dietrich U, Balfe P, et al. Genetic analysis of Human Immunodeficiency Virus type 1 and 2 (HIV-1 and HIV-2) mixed infections in India reveals a recent spread of HIV-1 and HIV-2 from a single ancestor for each of these viruses. J Virol 1994;68:2161-8.
4. Artenstein AW, VanCott TC, Mascola JR, et al. Dual infection with HIV type 1 of distinct envelope subtypes in humans. J Infect Dis 1995;171:805-10.
5. Gao F, Yue L, Robertson DL, et al. Genetic diversity of human immunodeficiency virus type 2: evidence for distinct sequence subtypes with differences in virus biology. J Virol 1994;68:7433-47.
6. Larsen N, Olsen GJ, Maidak BN, el al. The ribosomal database project. Nucleic Acids Res. 1993;21:3021-3.
